# Microwave-Assisted Reduction Technology for Recycling of Hematite Nanoparticles from Ferrous Sulfate Residue

**DOI:** 10.3390/ma18143214

**Published:** 2025-07-08

**Authors:** Genkuan Ren

**Affiliations:** 1 Key Laboratory of Process Analysis and Control of Sichuan Universities, Yibin University, Yibin 644000, China; 2004113001@yibinu.edu.cn; 2 College of Materials and Chemical Engineering, Yibin University, Yibin 644000, China

**Keywords:** microwave-assisted reduction technique, hematite nanoparticles, ferrous sulfate residue, pyrite

## Abstract

Accumulation of ferrous sulfate residue (FSR) not only occupies land but also results in environmental pollution and waste of iron resource; thus, recycling of iron from FSR has attracted widespread concern. To this end, this article shows fabrication and system analysis of hematite (HM) nanoparticles from FSR via microwave-assisted reduction technology. Physicochemical properties of HM nanoparticles were investigated by multiple analytical techniques including X-ray diffraction (XRD), Fourier transform infrared spectrum (FTIR), Raman spectroscopy, scanning electron microscopy (SEM), energy dispersive X-ray spectroscopy (EDX), transmission electron microscopy (TEM), X-ray photoelectron spectroscopy (XPS), ultraviolet visible (UV-Vis) spectrum, vibrating sample magnetometer (VSM), and the Brunauer–Emmett–Teller (BET) method. Analytic results indicated that the special surface area, pore volume, and pore size of HM nanoparticles with the average particle size of 45 nm were evaluated to be ca. 20.999 m^2^/g, 0.111 cm^3^/g, and 0.892 nm, respectively. Magnetization curve indicated that saturation magnetization Ms for as-synthesized HM nanoparticles was calculated to be approximately 1.71 emu/g and revealed weakly ferromagnetic features at room temperature. In addition, HM nanoparticles exhibited noticeable light absorption performance for potential applications in many fields such as electronics, optics, and catalysis. Hence, synthesis of HM nanoparticles via microwave-assisted reduction technology provides an effective way for utilizing FSR and easing environmental burden.

## 1. Introduction

Ferrous sulfate residue (FSR) is an industrial by-product from titanium dioxide produced via leaching ilmenite (FeTiO_3_) with concentrated sulfuric acid. As reported, about 3.0–4.0 tons of FSR were manufactured for producing 1.0 ton of titanium dioxide, and over 7.49 million tons of FSR in 2023 were generated from titanium dioxide production in China [1,2]. With the skyrocketing demand for titanium dioxide, the annual output of FSR persistently increases year after year [3,4,5]. Currently, only a small portion of FSR is recycled due to its high impurity content (e.g., MgSO_4_ and MnSO_4_) [6], and the rest is still treated as waste and stacked like a mountain. Even worse, the vast amount of FSR is accumulated, which not only causes severe environmental pollution, but also results in the massive waste of resources [7]. Hence, it is of critical importance to explore a cost-effective technique for the resource utilization of FSR. At present, extensive research has been conducted on the resource utilization of FSR, such as fabrication of magnesium doped magnetite nanoparticles [8], magnesium ferrite [9], nano α-Fe_2_O_3_ red pigment [10] and etc. Although these studies have offered some feasible approaches for recycling FSR, there are some limitations in practical application because of poor market requirements or high energy consumption. Thus, the sustainable utilization of FSR to obtain high-value chemical products is still a challenge.

Hematite (HM) nanoparticles have been widely applied in many fields such as adsorbing material, magnetic material, photocatalyst, and others [11,12,13] due to their strong corrosion resistance, nontoxicity, biocompatibility, environmental benignity, large surface area, and quantum dimension effect [14,15]. Currently, HM nanoparticles were fabricated using chemical reagents as crude materials, which has restricted their practical applications because of high production costs. To reduce production cost and enhance comprehensive utilization of FSR, HM nanoparticles fabricated using FSR as a starting material not only enhance sustainable utilization of FSR but also address the problem of pollution. To date, many techniques for the synthesis of HM nanoparticles have been reported, including coprecipitation [16], oxidation–precipitation [17], sol–gel technique [18], microemulsion [19], biosynthesis [20], solvothermal method [21], and hydrothermal method [22,23]. As compared with the aforementioned technologies, the reduction technique is better suited for the fabrication of HM nanoparticles due to its simple process, easy operation, mild reaction conditions, and no secondary pollution. But the electric heating method used in a previous study [24] has shortcomings of high energy consumption, long heating time, and slow heating rate. Furthermore, heating is transferred from the surface to the inside of the raw material to heat the material via conduction. The electric heating method inevitably forms a temperature gradient inside the material, resulting in local overheating of the raw material [25]. As for the microwave heating process, the heat is generated inside the raw material owing to the high-frequency reciprocating motion of dipole molecules inside particles. Therefore, microwave heating simultaneously heats the raw material inside and outside of the material [26,27,28]. Compared to the electric heating method, the microwave heating method has the properties of a fast heating rate, high energy efficiency, well-distributed heating, and low energy consumption [29]. Hence, microwave heating is used as a more effective and efficient method to fabricate HM nanoparticles.

Hematite nanoparticles were fabricated from FSR and pyrite with a microwave-assisted reduction technique under nitrogen protection. It is important to mention that the FSR and pyrite used in the synthesis process were, respectively, by-products of titanium dioxide production and copper concentrate production, and usually discarded. Therefore, there is no cost of raw materials in the synthesis process. Furthermore, SO_2_ emitted in the synthesize process was used to produce sulfuric acid, which can be compensated for insufficient production costs. Additionally, hematite nanoparticles synthesized from FSR and pyrite as raw materials reduced the construction cost of landfill. Thus, it can be seen that hematite nanoparticles fabricated from FSR and pyrite are feasible in both economic and environmental aspects.

The major objective of this study is to successfully fabricate HM nanoparticles from FSR via a microwave-assisted reduction technique. The physicochemical properties of HM nanoparticles fabricated by the microwave-assisted reduction technique were analyzed by X-ray diffraction (XRD), Fourier transform infrared spectrum (FTIR), Raman spectrum, scanning electron microscopy equipped with energy dispersive X-ray spectroscopy (SEM/EDX), transmission electron microscopy (TEM), Brunauer–Emmett–Teller method (BET), UV-Vis spectrophotometer, vibrating sample magnetometer, and X-ray photoelectron spectroscopy (XPS).

## 2. Experimental Section

### 2.1. Materials

Ferrous sulfate residue (FeSO_4_) was derived from Sichuan Province (Panzhihua, China). Pyrite (FeS_2_) was supplied from Sichuan Province (Leshan, China). Ferrous sulfate residue and pyrite were directly used without further refining. Anhydrous ethanol was of analytical grade, procured from Tianjin Kemiou Chemical Reagent Co., Ltd. (Tianjin, China). Deionized (DI) water was prepared using a three-level reverse osmosis membrane.

### 2.2. Fabrication of Hematite Nanoparticles

**Figure 1:**
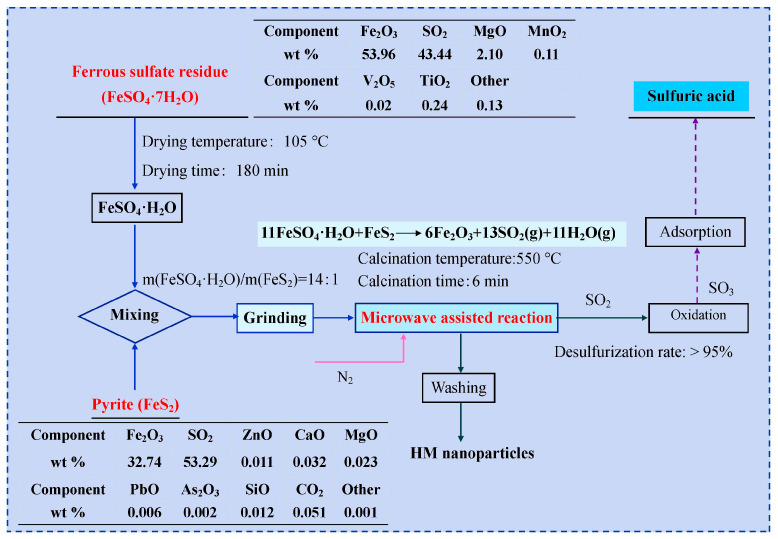
Schematic process flow diagram for the fabrication of HM nanoparticles.

HM nanoparticles were manufactured via a microwave-assisted reduction technique in nitrogen flow using FSR as an iron precursor. Firstly, the as-received FSR (FeSO_4_·7H_2_O) was transformed into ferrous sulfate monohydrate (FeSO_4_·H_2_O) by vacuum drying at 105 °C for 180 min. After that, ferrous sulfate monohydrate and pyrite were separately milled into an average diameter of less than 82 μm in an omnidirectional planetary ball mill with a 1:5 powder-to-ball ratio. Subsequently, ferrous sulfate monohydrate was mixed perfectly with pyrite at a mass ratio of 14:1 in an agate mortar. Next, well-mixed feedstock was put into a quartz boat and then transferred to the high temperature zone of the microwave tube furnace with programmed temperature control. After calcining at 550 °C for 6 min, the calcined specimens were naturally cooled to room temperature and washed repeatedly with anhydrous ethanol and DI water to remove soluble impurities. Finally, the resultant nanoparticles were dried at 80 °C for 360 min and used for performance analysis. Additionally, the desulfurization rate (SO_2_) of the feedstock during the reaction process was as high as 95%. Then, the SO_2_ discharged in the reaction process was converted to SO_3_ through catalytic oxidation and applied to produce sulfuric acid. The prepared sulfuric acid was applied in the production process of titanium dioxide with the sulphate process to achieve the recycling of sulfur resources. A schematic process flow diagram for the fabrication of HM nanoparticles is given in Figure 1.

### 2.3. Characterization

Crystal structure determination of the resulting sample was performed with an Empyrean X-ray diffractometer (XRD, PANalytical B.V, Almelo, The Netherlands) using Cu-Kα as the radiation source (λ = 1.5406 Å). Morphology and chemical composition of the resulting sample were investigated by scanning electron microscopy equipped with energy dispersive X-Ray spectroscopy (SEM/EDX, JSM7500F, JEOL, Akishima, Tokyo, Japan) and transmission electron microscopy (TEM, EM-2100Plus, JEOL, Akishima, Tokyo, Japan). Fourier transform infrared spectrum (FTIR, INVENIOR, Brooke Corporation, Karlsruhe, Germany) was applied for identifying the functional group of the sample. Raman spectroscopy (RM, Lab-RAM, HORIBA, Paris, France) was performed at ambient temperature with an excitation wavelength of 633 nm. Surface elemental constitution and valence state of the resulting sample were analyzed by X-ray photoelectron spectroscopy (XPS, AXIS-Supra, Kratos, Manchester, UK), and the C1s peak at 284.8 eV was used as a reference value for calibration. The measurements of surface area, pore size, and pore volume of the resulting sample were conducted via nitrogen adsorption/desorption isotherms according to the Brunauer–Emmett–Teller method at 77 K (BET, Quan-tachrome, Boynton Beach, FL, USA).

## 3. Results and Discussion

### 3.1. Crystal Structure

Ferrous sulfate residue typically contains impurities such as MgSO_4_ and MnSO_4_. Different substances react with pyrite in different ways. The temperature of spontaneous reactions varies.

**Figure 2:**
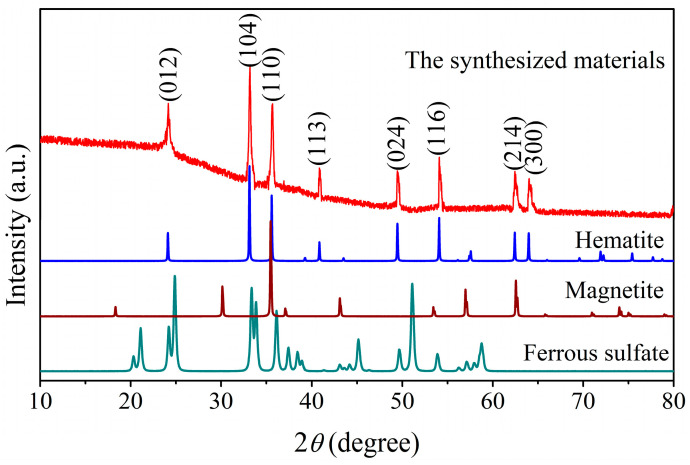
X-ray diffraction pattern for the synthesized hematite nanoparticles.

To determine the crystalline structure of the as-prepared sample, the as-prepared sample was investigated by the XRD technique, and the corresponding XRD pattern is displayed in Figure 2. As shown in Figure 2, the observed diffraction peaks at 2*θ* of 24.0°, 33.1°, 35.6°, 40.8°, 49.4°, 54.0°, 62.4°, and 64.1° respectively correspond with the lattice planes of (012), (104), (110), (113), (024), (116), (214), and (300) of hematite with the rhombohedral structure. These diffraction peaks are completely in line with the diffraction peaks of the standard hematite (JCPDS 33-0664), revealing that the main crystalline structure of the as-prepared nanoparticles is hematite. A small diffraction peak observed at 36.95° corresponds to the standard diffraction peak of MgO (JCPDS 87-0652), which can be attributed to MgO in HM nanoparticles. Additionally, the crystallite size estimated from the (104) lattice plane based on the Scherrer equation is found to be ca. 41 nm.

### 3.2. FTIR and RM Analysis

**Figure 3:**
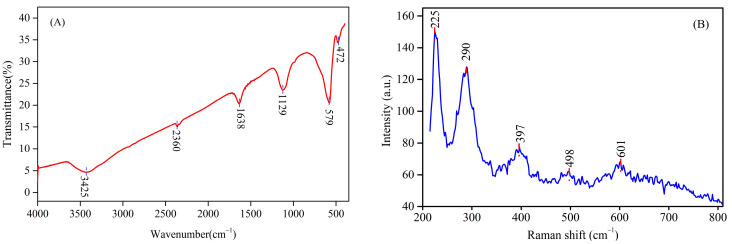
(**A**) FTIR spectrum and (**B**) Raman spectrum of HM nanoparticles (reaction temperature: 550 °C; reaction time: 6 min; FeSO_4_/FeS_2_: 14:1).

The Fourier transform infrared spectrum (FTIR) of materials prepared via the microwave-assisted reduction technique is depicted in Figure 3A. The two absorption peaks observed at about 3425 cm^−1^ and 1638 cm^−1^ correspond to the H-O-H stretching and bending modes of the adsorbed water moisture on the surface of nanoparticles, respectively [30]. Moreover, the characteristic band situated at approximately 1129 cm^−1^ refers to the C-O stretching vibration of anhydrous ethanol [31,32], which originates from the sample preparation process. The weak peak near 2360 cm^−1^ is attributed to the C=O stretching vibration of carbon dioxide adsorbed on the surface of particles [33]. The prominent peaks centered at 475 cm^−1^ and 579 cm^−1^ are separately ascribed to the characteristic stretching vibration of Fe-O in HM nanoparticles, which is in accordance with those of hematite in the literature [34]. Thus, the existence of these peaks verifies that hematite nanoparticles are successfully fabricated by the microwave-assisted reduction technique.

Raman spectroscopy was conducted to further support above the results. The Raman spectrum of the fabricated HM nanoparticles is displayed in Figure 3B. The synthesized HM belongs to the hexagonal structures with D_3d_^6^ space group, which has seven active phonon modes, namely, two external modes 2E_g_ and five internal modes (2A_1g_ + 3E_g_) [35]. As noted in Figure 3B, five phonon modes are identified at 225 cm^−1^, 290 cm^−1^, 397 cm^−1^, 498 cm^−1,^ and 601 cm^−1^, which can be assigned to internal modes 2A_1g_ + 3E_g_. The peaks located at 225 cm^−1^ and 498 cm^−1^ can be attributed to the Fe^3+^-O^2−^ stretching vibration mode A_1g_ [36], while the peaks that appeared at 290 cm^−1^, 397 cm^−1^, and 601 cm^−1^ are related to the symmetric bending modes E_g_, which are typical characteristic of hematite [37,38]. In addition to this, no characteristic peaks of iron oxides and iron oxyhydroxides are detected, which manifests the high purity of HM nanoparticles [16].

### 3.3. Chemical States

**Figure 4:**
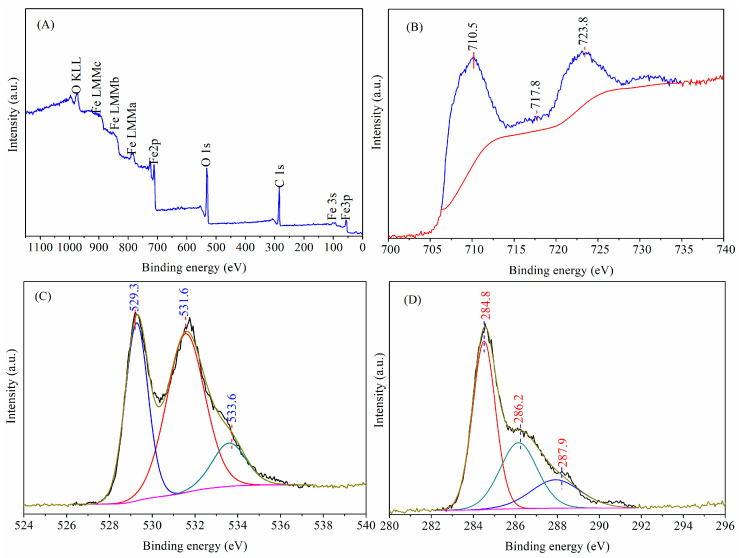
The XPS spectra of HM nanoparticles: (**A**) wide scan, (**B**) Fe2p, (**C**) O1s, and (**D**) Cls. (reaction temperature: 550 °C; reaction time: 6 min; FeSO_4_/FeS_2_: 14:1).

The XPS technique was implemented to investigate surface element valence states as well as chemical compositions of the manufactured HM nanoparticles by the microwave-assisted reduction technique. The XPS wide-scan survey spectrum and high-resolution spectrum of individual elements for the as-resulted HM nanoparticles are shown in Figure 4. As seen in Figure 4A, the signals of iron, oxygen, and carbon elements are detected in the wide-scan survey spectrum, where carbon is ubiquitous and attributed to hydrocarbon coming from the XPS instrument itself and atmospheric carbon dioxide loaded on the surface of HM nanoparticles [39]. The results indicate that the as-formed HM nanoparticles are mainly composed of iron and oxygen elements, which are in line with those of the EDX spectrum, as discussed later. The high-resolution C1s peak is fitted into three independent peaks, whose binding energies are located at 284.8 eV, 286.2 eV, and 287.9 eV, which can be respectively ascribed to lattice carbon, hydrocarbon, and atmospheric carbon dioxide. Meanwhile, the peak at 284.8 eV is chosen as a reference peak for calibration [40]. The high-resolution Fe2p spectrum of the as-synthesized HM nanoparticles shows the doublet peaks centered at the binding energies of 710.5 eV and 723.8 eV, separately attributed to spin-orbits of Fe2p_3/2_ and Fe2p_1/2_ with the doublet separation energy at 13.3 eV, which are consistent with those of hematite nanoparticles reported in the published literature [41]. In addition, the shakeup satellite peak centered at 717.8 eV is a typical characteristic of Fe^3+^ valence state, which confirms the Fe^3+^ oxidation state of iron in HM nanoparticles (Figure 4B) [34,42,43,44]. The O1s core region of HM nanoparticles can be deconvoluted into three individual peaks, whose binding energies are centered at around 529.3 eV, 531.6 eV, and 533.6 eV, respectively (Figure 4C). The characteristic peak centered at 529.3 eV is assigned to the lattice oxygen (Fe-O) in HM nanoparticles [32], whereas the characteristic peak at 531.6 eV is attributed to the O-H of surface adsorbed water molecules [36]. The peak observed at 533.6 eV may be caused by carbon dioxide adsorbed on the surface of HM nanoparticles. Therefore, the above results further verify that the prepared materials are hematite nanoparticles, as evidenced from the XRD as well as FTIR results. Additionally, the XPS wide-scan survey spectrum for HM nanoparticles prepared by the microwave heating is similar to that for hematite nanoparticles synthesized via the electric heating method in the previous study [24], which further confirms that the synthesized material is hematite nanoparticles. But it can be seen that the corresponding peaks observed from Fe2p, O1s, and C1s spectra shift in different degrees, which may be caused by differences in the composition of the synthetic materials. Meanwhile, the S2p peak in the XPS wide-scan survey spectrum disappears for HM nanoparticles prepared by the microwave heating method, indicating that ferrous sulfate reacts more thoroughly with pyrite. This attests that microwave heating has a better heating effect than electric heating.

### 3.4. Microstructure and Element Composition

**Figure 5:**
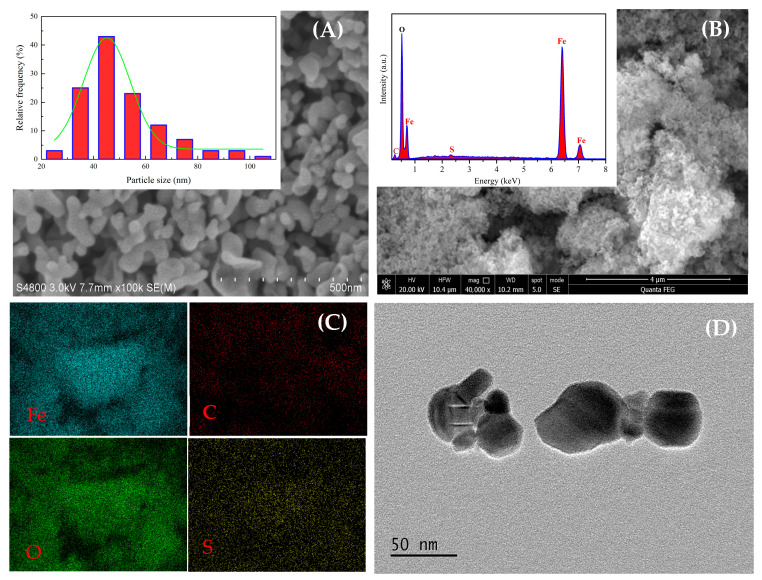
(**A**) SEM image, (**B**) EDX pattern, (**C**) EDX element mapping, (**D**) TEM image of HM nanoparticles (reaction temperature: 550 °C; reaction time: 6 min; FeSO_4_/FeS_2_: 14:1).

The particle diameter and morphology for the as-fabricated HM nanoparticles were investigated in detail by SEM and TEM, as shown in Figure 5. It can be clearly seen from the SEM image that the as-resulted HM nanoparticles are primarily composed of nanoparticles of spherical-like morphology in structure. The TEM image indicates that the synthesized HM nanoparticles show porous structures composed of smaller nanosize particles, which can be ascribed to sulfur dioxide emitted from the reaction process, as verified by nitrogen adsorption analysis. The particle size distribution obtained from the SEM image by counting ca.100 nanoparticles using Image J 1.8.0 software is shown in Figure 5A. As can be noted from the curve of particle size distribution, the particle diameter of HM nanoparticles is mainly within the range of 35–80 nm with an average particle diameter of ~45 nm, which is in good agreement with that reckoned from XRD patterns. On the other hand, the divergence in particle sizes originates from agglomeration of particles due to intergranular electrostatic attraction and magnetic dipole interaction [45]. Meanwhile, the EDX spectrum was a very useful technique to analyze chemical composition and elemental distribution of the as-prepared HM nanoparticles. As observed in Figure 5B, the EDX spectrum of the resulting HM nanoparticles exhibits the corresponding peaks of C, O, Fe and S atoms, which is an indication that the as-fabricated HM nanoparticles contain abundant amounts of iron and oxygen, and trace amounts of sulfur. The carbon peak in the EDX spectrum is caused by the carbon copper grid applied in the process of analysis. Hence, these results further attest that the as-fabricated HM nanoparticles are principally composed of Fe and O elements. As observed from the EDX mapping (Figure 5C), all the elements are uniformly distributed in the whole spherical structure of HM nanoparticles.

### 3.5. Specific Surface Area and Pore Size

**Figure 6:**
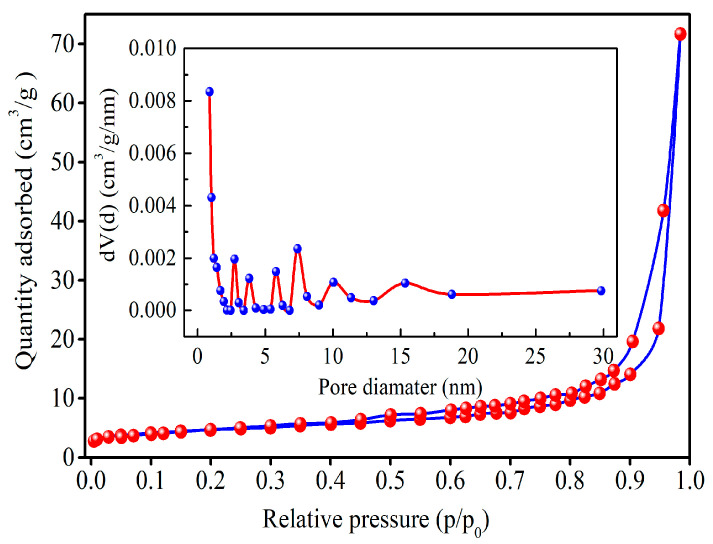
N_2_ adsorption/desorption isotherms and (insert) pore size distribution curve of HM nanoparticles (reaction temperature: 550 °C; reaction time: 6min; FeSO_4_/FeS_2_: 14:1).

BET (Brunauer–Emmett–Teller) analysis was applied to determine the specific surface area and pore size of the resultant HM nanoparticles. Nitrogen adsorption/desorption isotherms and pore size distribution of HM nanoparticles are depicted in Figure 6. Nitrogen adsorption/desorption isotherms on HM nanoparticles at low temperature show the combined features of type I and IV isotherm with H3-type hysteresis loop based on the IUPAC classification at the relative pressure range of 0.45–1.0, indicating the coexistence of micropores and mesopores in HM nanoparticles [46]. In addition, specific surface area is calculated to be ca. 20.997 m^2^/g with the BET method, which is higher than those of the reported literature [24]. Moreover, it is observed that the pore size distribution curve derived from the desorption branch of the isotherm based on the BJH method exhibits a multimodal shape, with the existence of contributions from micropores (up to 2 nm) and mesopores (between 2 nm and 30 nm). Mean pore size and pore volume of HM nanoparticles based on the BJH method are found to be ca. 0.892 nm and ca. 0.111 cm^3^/g, respectively. Hence, the results show that the as-fabricated HM nanoparticles belong to porous materials.

### 3.6. UV-Vis Spectrum

**Figure 7:**
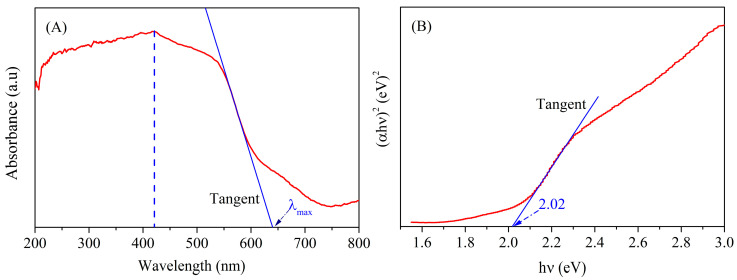
(**A**) UV-Vis absorbance spectrum of HM nanoparticles and (**B**) plot of (ahν)^2^ versus hν.

To appraise the optical performances of materials fabricated using microwave-assisted reduction technology, UV-vis spectroscopy analysis was performed as given in Figure 7A. Evidently, the prepared nanoparticles exhibit noticeable light absorption within the wavelength range of 200 nm–639 nm, whose absorption threshold falls into the visible light region. Thus, it can be seen that the fabricated nanoparticles using ferrous sulfate residue as an iron source may be used as a visible light-responsive catalyst. The as-prepared nanoparticles have broader absorption range as compared with hematite in literature [47,48], which may be due to the effect of impurities (Ti, Mg, Al) in FSR. Additionally, the maximum absorption peak in the UV-Vis absorption spectrum is close to 421 nm. As compared with the traditional heating method [24], the maximum absorption peak of HM nanoparticles formed via the microwave-assisted heating method demonstrates obvious red-shifts from the ultraviolet region (360 nm) to the visible light region (421 nm), indicating that the microwave-assisted heating method enhances absorption light capacity in the visible light region.

The band gap energy (*E_g_*) for HM nanoparticles is estimated based on the Tauc Formula (1) [49].

(1) $${\left(\alpha hv\right)}^{n}=A{\left(hv-E_{g}\right)}^{2}$$

where *ɑ* and *hν* respectively represent the absorption coefficient and photon energy, *A* denotes a constant related to valence and conduction bands of specific materials, *n* shows conversion characteristics, and *n* is 2 for an indirect transition. The Tauc’s plot obtained from UV–visible data according to Formula (1) is displayed in Figure 7B. As shown in Figure 7B, the band gap energy of HM nanoparticles is calculated to be 2.02 eV, which is smaller than those of hematite in the reported literature [47,50]. This may be due to that the impurities (Mg, Al) in HM nanoparticles from FSR introducing impurity energy levels and decreasing the band gap energy.

### 3.7. Magnetic Properties

**Figure 8:**
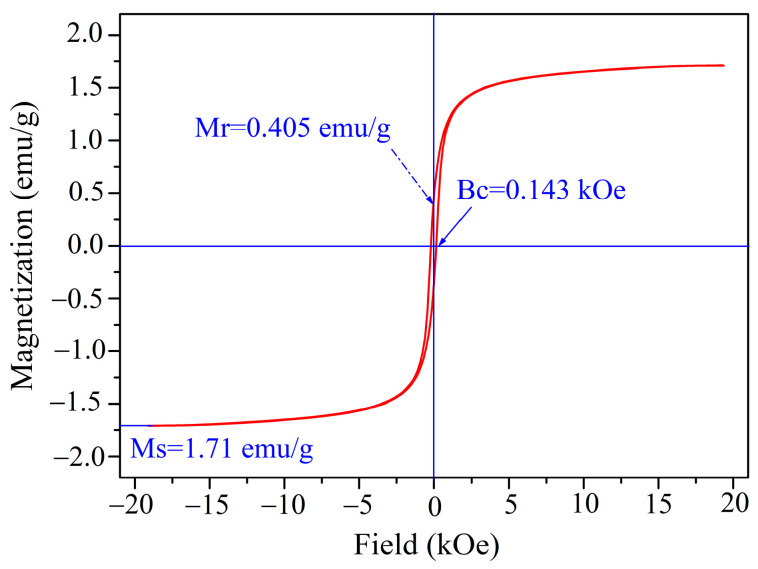
Magnetization curve of the synthesized HM nanoparticles.

To further evaluate the magnetic properties of as-synthesized specimens, the room-temperature magnetization curve of as-synthesized specimens was investigated using a vibrating sample magnetometer in the magnetic field ranging from −20 kOe to 20 kOe, as displayed in Figure 8. From Figure 8, magnetization curve of as-prepared materials demonstrated the hysteresis behavior with low coercivity (H_c_) of 143 Oe and remanent magnetization (M_r_) of 0.405 emu/g, indicating that the fabricated nanoparticles reveal weakly ferromagnetic characteristics at room temperature. The measured value of saturation magnetization Ms for as-synthesized hematite is calculated to be approximately 1.71 emu/g, which is higher than those for hematite in the literature [51]. Herein, the obtained high Ms value verifies the superparamagnetic behavior of synthesized hematite nanoparticles, which is one of the characteristics of good magnetic material. Low coercivity is principally owing to the equiaxed hematite nanoparticles and their anisotropy and the disorder of the crystal axis [52].

## 4. Conclusions

Hematite (HM) nanoparticles have been successfully fabricated using FSR as the starting material via the microwave-assisted reduction technique at slow nitrogen flow. The XRD analysis showed that the main crystalline structure of as-prepared nanoparticles is hematite with the rhombohedral structure of HM nanoparticles at 550 °C for 6 min. The XPS analysis further certifies that the fabricated materials were high-purity hematite nanoparticles. The SEM and TEM images confirmed that the resultant HM nanoparticles showed spherical-like morphology with an average particle size of ~45 nm. BET surface area, pore volume, and pore size of HM nanoparticles were separately estimated to be ca. 20.997 m^2^/g, 0.111 cm^3^/g, and 0.892 nm. Additionally, the absorption threshold of the prepared nanoparticles falls into the visible light region, and the prepared nanoparticles may be used as visible-light-responsive catalysts. The measured value of saturation magnetization Ms for as-synthesized hematite is calculated to be approximately 1.71 emu/g. Furthermore, the synthesized materials revealed porous structures composed of smaller nanosize particles, as confirmed by TEM and BET. As a result, the above results demonstrate that the synthesized HM nanoparticles by the microwave-assisted reduction route have promising application prospects for adsorbing materials in many fields.

## Data Availability

The original contributions presented in this study are included in the article/Supplementary Materials. Further inquiries can be directed to the corresponding author.

## Supplementary Materials

The following supporting information can be downloaded at: https://www.mdpi.com/article/10.3390/ma18143214/s1.
